# ASNS disruption shortens CTPS cytoophidia in *Saccharomyces cerevisiae*

**DOI:** 10.1093/g3journal/jkaa060

**Published:** 2021-01-11

**Authors:** Shanshan Zhang, Han-Chao Feng, Ji-Long Liu

**Affiliations:** 1 School of Life Science and Technology, ShanghaiTech University, Shanghai, 201210, China; 2 University of Chinese Academy of Sciences, Beijing, 100049, China; 3 Shanghai Institute of Biochemistry and Cell Biology, Chinese Academy of Sciences, Shanghai, 200031, China; 4 Department of Physiology, Anatomy and Genetics, University of Oxford, Oxford, OX1 3PT, UK

**Keywords:** asparagine synthetase, CTP synthase, cytoophidium, glutamine metabolism, *Saccharomyces cerevisiae*

## Abstract

Asparagine synthetase (ASNS) and CTP synthase (CTPS) are two metabolic enzymes that catalyze the biosynthesis of asparagine and CTP, respectively. Both CTPS and ASNS have been identified to form cytoophidia in *Saccharomyces cerevisiae*. Glutamine is a common substrate for both these enzymes, and they play an important role in glutamine homeostasis. Here, we find that the ASNS cytoophidia are shorter than the CTPS cytoophidia, and that disruption of ASNS shortens the length of CTPS cytoophidia. However, the deletion of CTPS has no effect on the formation and length of ASNS cytoophidia, or on the ASNS protein level. We also find that Asn1 overexpression induces the formation of a multi-dot structure in diauxic phase which suggests that the increased protein level may trigger cytoophidia formation. Collectively, our results reveal a connection between ASNS cytoophidia and CTPS cytoophidia.

## Introduction

In 2010, three laboratories independently reported that CTP synthase (CTPS) assembles into filamentous structures termed cytoophidia in bacteria, yeast, and fruit fly (Ingerson-Mahar *et al.* 2010; Liu 2010; Noree *et al.* 2010). These newly discovered membraneless structures formed by metabolic enzymes were found to be evolutionarily conserved (Chen *et al.* 2011; Azzam and Liu 2013; Barry *et al.* 2014; Aughey *et al.* 2016; Liu 2016; Daumann *et al.* 2018; Andreadis *et al.* 2019; Sun and Liu 2019a, 2019b; Wu and Liu 2019; Zhang and Liu 2019; Zhou *et al.* 2019; Zhang *et al.* 2020a, 2020b; Zhou *et al.* 2020). To date, the understanding of cytoophidia has been greatly expanded by genome-wide screening in *Saccharomyces cerevisiae* and it has been observed that most metabolic enzymes form filamentous or punctuated cytoophidia (Shen *et al.* 2016; Noree *et al.* 2019a). These enzymes cover a variety of biological reactions, such as glucose metabolism, translation initiation mechanisms, and purine biosynthesis (Shen *et al.* 2016; Noree *et al.* 2019a).

Asparagine synthetase (ASNS) catalyzes ATP-dependent de novo biosynthesis of asparagine from aspartic acid, and it has been observed to form cytoophidia in budding yeast. In *Escherichia coli*, there are two types of ASNS, glutamine dependent and ammonia dependent (Humbert and Simoni 1980; Ramos and Wiame 1980). *ASN1* and *ASN2* both code glutamine-dependent ASNS in budding yeast. Only a double mutant of *ASN1* and *ASN2* will lead to total auxotrophy (Jones 1978; Ramos and Wiame 1980; Dang *et al.* 1996). Asn1 and Asn2 have high similarity in terms of protein size and amino acid sequence. The capabilities of Asn1 and Asn2 in cytoophidia formation are distinct, and Asn2 cytoophidium formation is dependent on the presence of Asn1 (Zhang *et al.* 2018; Noree *et al.* 2019b).

CTP is a precursor of DNA and RNA biosynthesis and it participates in nucleotide metabolism and membrane phospholipid biosynthesis. The rate-limiting step of de novo biosynthesis of CTP is catalyzed by CTPS. *URA7* and, to a lesser extent, *URA8* are the genes that code for CTPS in *S. cerevisiae* (Ozier-Kalogeropoulos *et al.* 1994), and Ura7 and Ura8 are very similar in protein size and amino acid sequence. The maximum expression of CTPS is observed in the exponential phase (Nadkarni *et al.* 1995). It has been proven that CTPS cytoophidium formation in bacteria inhibits the enzymatic activity (Barry *et al.* 2014), whereas in human cells CTPS1 cytoophidium formation increases the enzymatic activity (Lynch *et al.* 2017). *Drosophila* CTPS can form conformationally different substrate-bound and product-bound filaments (Zhou *et al.* 2019). CTPS2 cytoophidium conformation can switch between active and inactive forms based on the substrate and product levels (Lynch and Kollman 2020). These research results support that cytoophidium formation provides an additional layer of metabolism regulation.

The spatial relationship between ASNS and CTPS cytoophidia has been observed as a head-to-head or side-by-side pattern (Zhang *et al.* 2018). To gain a better understanding of the association between these two types of cytoophidia, we explore the effect of ASNS deletion on CTPS cytoophidia and vice versa. We also study changes in ASNS and CTPS cytoophidia in response to different culture media.

## Materials and methods

### Yeast strains and culture media

Yeast strains used in this study were derived by transformation of BY4741 (donated by Jinqiu Zhou from Shanghai Institute of Biochemistry and Cell Biology). Transformation was performed by the lithium acetate method. The generation of *URA7-GFP ASN1-MCHERRY* and *ASN1-GFP ASN2-MCHERRY* cells has been described in our previous study (Zhang *et al.* 2018). The genotypes of all strains are listed in Table 1. *Saccharomyces cerevisiae* cells were cultured in standard rich medium (YPD: 2% peptone, 1% yeast extract, 2% dextrose) or YPG (2% peptone, 1% yeast extract, 2% galactose) at 30°C unless otherwise indicated.

**Table 1:** *Saccharomyces cerevisiae* strains used in this study

Strain name	Genotype
*BY4741*	*MATα his3Δ leu2Δ ura3Δ0 met15Δ*
*Ura7-GFP Asn1-mCherry*	*As BY4741, ura7-GFP(His3MX6), asn1-mCherry(KanMX6)*
*Asn1-GFP Asn2-mCherry*	*As BY4741, asn1-GFP(His3MX6), asn2-mCherry(KanMX6)*
*Ura7-GFP Gal-Asn1-mCherry*	*As BY4741, ura7-GFP(His3MX6), asn1-mCherry(KanMX6) P_GAL10_-ASN1*
*Gal-Asn1-GFP Asn2-mCherry*	*As BY4741, asn1-GFP(His3MX6), asn2-mCherry(KanMX6) P_GAL10_-ASN1*
*Ura7-GFP Asn1△*	*As BY4741, ura7-GFP(His3MX6), asn1::URA3*
*Ura7-GFP Asn2△*	*As BY4741, ura7-GFP(His3MX6), asn2::URA3*
*Asn1-GFP Ura7△*	*As BY4741, Asn1-GFP(His3MX6), ura7::URA3*
*Ura7^WT^-GFP*	*As BY4741, ura7-GFP(His3MX6)*
*Ura7^H360A^-GFP*	*As BY4741, ura7^H360A^-GFP(His3MX6)*

### Gene disruption

*ASN1* and *ASN2* disruption strains containing *URA7-GFP* were obtained by PCR-based gene targeting using pRS303 and pRS306 as described previously (Zhang *et al.* 2018). For *URA7* disruption cassette construction, the 5’ untranslated region and 3’ region were sub-cloned into pRS306. The disruption cassettes first linearized with *EcoR I* (New England Biolabs) and transformed into *ASN1-GFP* cells to construct *ASN1-GFP URA7△* strains. All the primers used for *URA7* gene deletion are listed in Table 2. Single colonies obtained by transformation were screened by PCR to confirm the absence of the specific gene.

**Table 2:** Primers used for *URA7* gene disruption

Primer name	Sequence (5′ to 3′)
URA7 UHA-F	ggg**GAATTC**TTAAAGTTAGCCCTCCCATCTT
URA7 UHA-R	ggg**GGGTACC**GTTCTATTGACCAATTCACT
URA7 DHA-F	ggg**TCTAGA**ATATTTGTAGTGCTTCTCTACAC
URA7 DHA-R	ggg**GAATTC**TGATAAATAATCTCCCTGTTCA
URA7 KO testing F	AACCACCTGTACGAACTGGCAC
URA7 KO testing R	CCAGTTGAAGATGCAAGAACAC

### *ASN1* overexpression

To construct *ASN1* overexpression strain, *ASN1* in *URA7-GFP ASN1-MCHERRY* and *ASN1-GFP ASN2-MCHERRY* cells was manipulated by transformation of a linear PCR product consisting of GAL1 promoter sequence with 40 bp of flanking sequences upstream of the ASN1 coding sequence. *pFA6a-LEU-GAL1* plasmid originated from *pFA6a-TRP-GAL1* plasmid was used for GAL sequence amplification. Primers used for *pFA6a-LEU-GAL1* plasmid construction and *ASN1* overexpression strain construction are, respectively, listed in Tables 3 and 4. Transformants were selected on SD+Leu and positive clones were validated by sequencing. The expression of Asn1 was driven by endogenous GAL promoter which was induced when cells were grown on galactose.

**Table 3:** Primers used for *pFA6a-Leu-GAL1* plasmid construction

Primers	Sequences (5′ to 3′)
Leu F	CTGATATCATCGATGAATTCATTGCGTATATA GTTTCGTCTACC
Leu R	ATGGGGCTCTTTACAGATCTAACTGTGGGAATA CTCAGGT
Leu t F	CTGATCGCATACTCTTCTTACC
Leu t R	TAAGACCATGTAACTTTGCA

**Table 4:** Primers used for *ASN1* overexpression strain construction

Primer name	Sequence (5ʹ to 3ʹ)
Asn1 OE F	GCACGTCTTCGTGCCTGAAAGCGGCGAAAATA CCACACATTTTGAGATCCGGGTTTT
Asn1 OE R	CTTGCTTTACGCTAAGGATATAAATCGGACGT AACTTAAGCGCATAGGCCACTAGTGGAT
Asn1 OE t F	CTGATCGCATACTCTTCTTACC
Asn1 OE t R	GCATTACCGGACCAATCTG
Asn1 OE S1	GAAGAACCTCAGTGGCAAAT

### Glutamine analog treatment

A glutamine analog 6-diazo-5-oxo-L-norleucine (DON) was used to treat *ASN1-GFP ASN2-mCherry* cells. We cultured the cells in standard rich medium (YPD) with 0%, 2%, and 4% DON content, respectively. After 7 days of culture, when the cells were at stationary phase, they were collected and fixed with 4% PFA.

To count the abundance of cytoophidia, more than 1000 cells were counted manually for each strain in each trial. Three groups of trials were mainly carried out manually. To count the length of cytoophidia, more than 200 cells were counted manually for each strain in each of the three biological repeats. The experimental results were processed by ANOVA function of software GraphPad Prism. *P < 0.05, **P < 0.01, and ***P < 0.001.

### Microscopy

Cells were first fixed by 4% paraformaldehyde (PFA) after culture for the indicated time at room temperature for 10 min (Zhang *et al.* 2021). Then cells were washed once with sterile water before being mixed with PBS mountant (1.2% LMT agarose in PBS). Images for cytoophidium abundance and length quantification were captured using a Zeiss Axio Imager 2 microscope and Zeiss LSM 880 inverted laser-scanning confocal microscope, respectively. Then these images were processed with ZEN 2 lite (blue edition) and ImageJ Fiji software.

### Data availability

Strains and plasmids are available upon request. The authors affirm that all data necessary for confirming the conclusions of the article are present within the article, figures, and tables.

## Results

### Comparison between Ura7 and Asn1 cytoophidia

**Figure 1:**
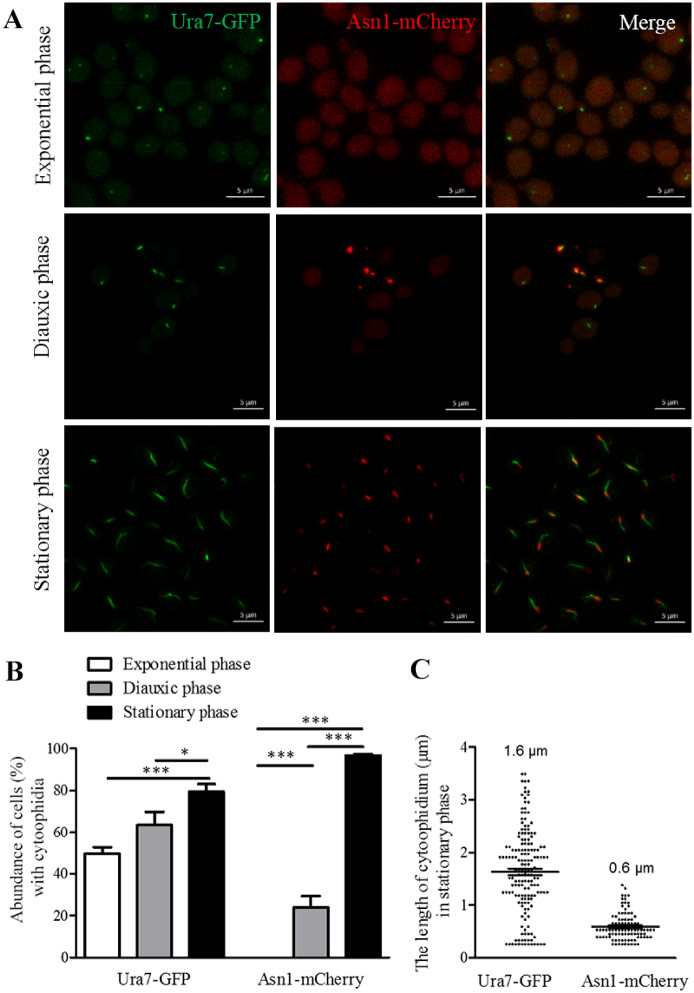
Ura7 and Asn1 cytoophidia in *S. cerevisiae*. Ura7-GFP Asn1-mCherry cells were grown in rich medium and cells were collected after 6, 24, and 168 h culture. The cells were subsequently fixed by 4% PFA at room temperature for 10 min. Ura7-GFP and Asn1-mCherry protein were observed by fluorescent microscopy. The abundance of cytoplasmic cytoophidia were calculated and the average length of cytoplasmic cytoophidia were plotted. (A) Representative confocal image of Ura7-GFP Asn1-mCherry cells. Ura7 cytoophidia were observed in all three growth phases. Scale bar 5 μm. (B) Quantification of cells with visible cytoophidia was plotted and expressed as percentage of cells containing cytoophidia in three growth phases. *P < 0.05 and ***P < 0.001 (C) The average length of Ura7 and Asn1 cytoophidia was measured and plotted in the stationary phase.

In our previous research, we observed that in most cells Ura7 cytoophidia and Asn1 cytoophidia overlapped in the middle or at the ends (Zhang *et al.* 2018). For further study, we generated the double tagging strain Ura7-GFP Asn1-mCherry. Cells of this strain were collected in three growth phases: exponential phase, diauxic phase and stationary phase (Figure 1A). The pattern of cytoophidium formation of these two metabolic enzymes is similar, in general. With cell growth, the abundance of Asn1 and Ura7 cytoophidia gradually increases and reaches a peak in the stationary phase (Figure 1B). In the exponential phase, dot-shaped Ura7 cytoophidia were observed. However, there are differences: Ura7 starts to form cytoophidia in the exponential phase and for Asn1 it starts in the diauxic phase (Figure 1, A and B). Ura7 cytoophidia are crooked and Asn1 cytoophidia are short and rod shaped in the stationary phase (Figure 1B). The length quantification results showed that the average length of Ura7 cytoophidia and Asn1 cytoophidia is 1.6 μm and 0.6 μm, respectively.

### Either Asn1 or Asn2 disruption shortens Ura7 cytoophidia

**Figure 2:**
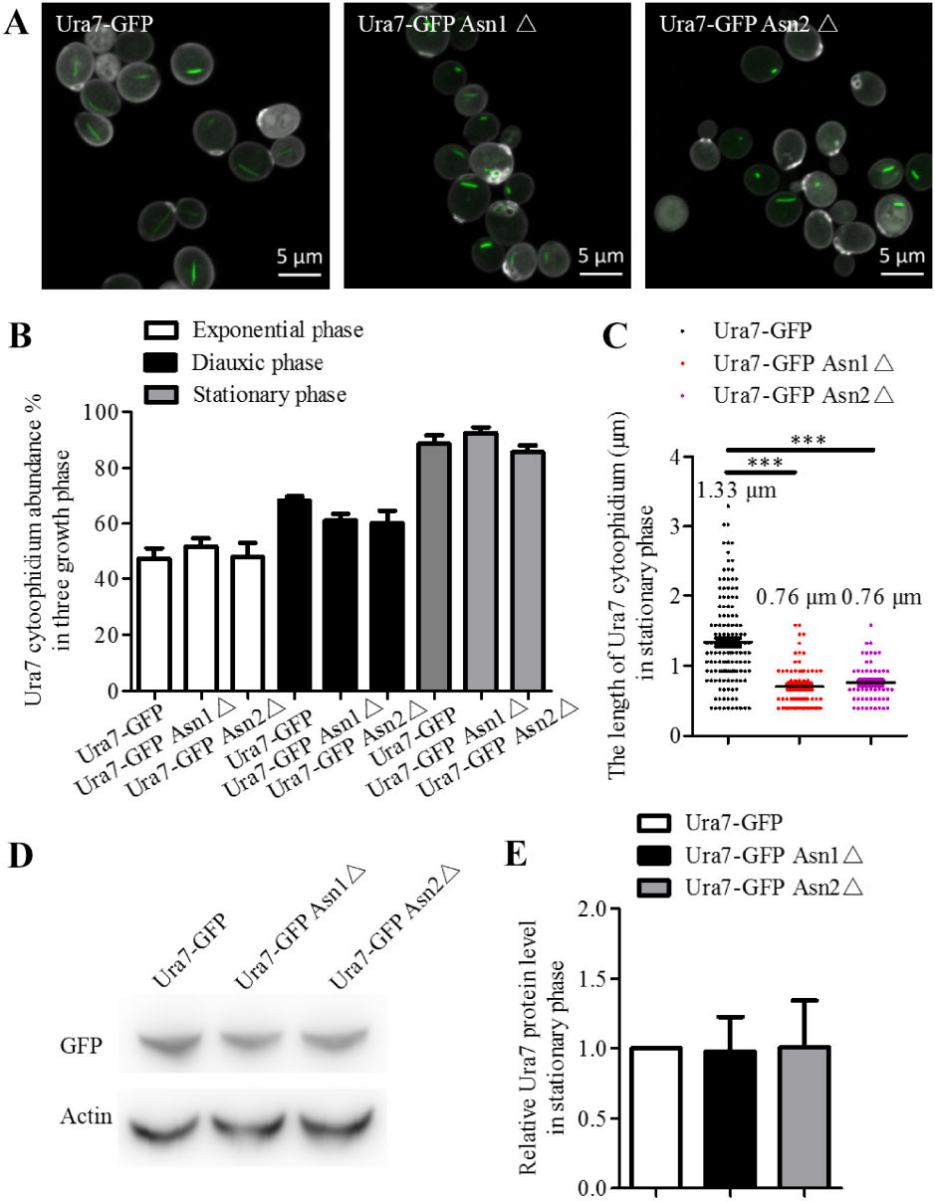
The effect of Asn1 or Asn2 disruption on Ura7 cytoophidia. Cells were grown in rich medium and collected in exponential, diauxic and stationary phase. The cells were subsequently fixed by 4% PFA at room temperature for 10 min. Ura7-GFP and Asn1-mCherry protein were observed by fluorescent microscopy. (A) Representative images of *URA7-GFP, URA7-GFP ASN1△ AND URA7-GFP ASN2△* strain in stationary phase and details of Ura7 cytoophidium morphology. Scale bar 5 μm. (B) Quantification of cells with visible Ura7 cytoophidia was plotted and expressed as percentage of cells containing cytoophidia in *URA7-GFP, URA7-GFP ASN1△ AND URA7-GFP ASN2△* strains from exponential phase to stationary phase. (C) Ura7 cytoophidium length analysis of *URA7-GFP, URA7-GFP ASN1△ AND URA7-GFP ASN2△* strains at stationary phase. ***P < 0.001. (D) Western blot analysis of Ura7 protein level in *URA7-GFP, URA7-GFP ASN1△ AND URA7-GFP ASN2△* strains at stationary phase. (E) Protein levels were plotted after normalization over alpha-actin levels and the protein level of Ura7 in Ura7-GFP cells is referred to as 1.

To have a clear understanding of the relationship between Ura7 and Asn1 cytoophidia, we tried to determine whether Ura7 cytoophidia are affected when Asn1 or Asn2 is disrupted. Hence, we generated Ura7-GFP Asn1△ and Ura7-GFP Asn2△ strains based on the Ura7-GFP strain (Figure 2A). The analysis of Ura7 cytoophidia in these three strains showed that there was no significant change in abundance in the three growth phases (Figure 2B). However, the average length of Ura7 was dramatically decreased when either Asn1 or Asn2 was disrupted. Before the ASNS disruption, the Ura7 cytoophidia had a wider length range, and average length was 1.33 μm. There were cytoophidia more than 2 μm long and we even observed some of more than 3 μm. After either Asn1 or Asn2 disruption, the average length of the Ura7 cytoophidia was 0.76 μm and all were less than 2 μm in length (Figure 2C). We also compared the Ura7 protein levels in Ura7-GFP, Ura7-GFP Asn1△ and Ura7-GFP Asn2△ cells to check whether the effect of ASNS disruption results from a change in protein level, but there was no significant difference between the three strains (Figure 2, D and E). ASNS disruption therefore affects Ura7 cytoophidium length rather than its protein level in the stationary phase.

### Ura7 knockout has no obvious effect on Asn1 cytoophidia

**Figure 3:**
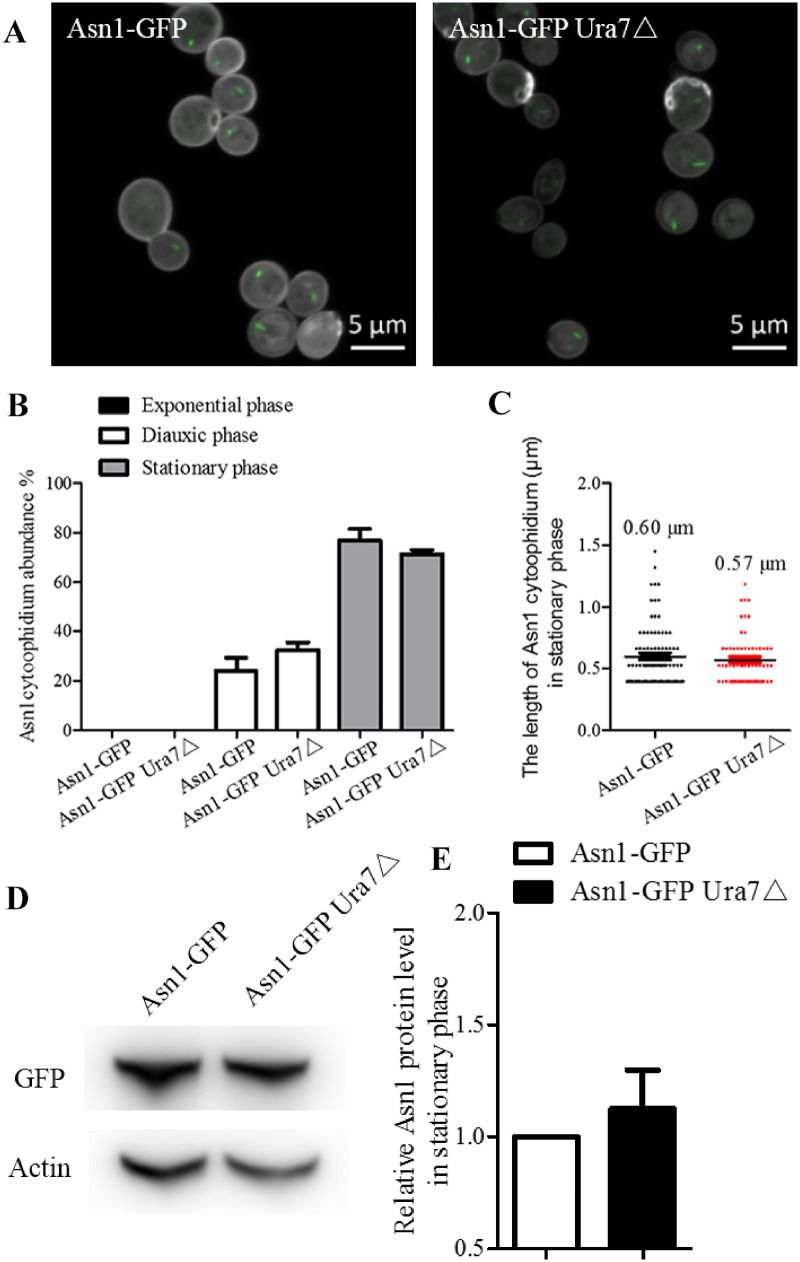
The effect of Ura7 disruption on Asn1 cytoophidia. Cells grown in rich medium were collected during three growth phases and observed by fluorescence microscopy. (A) Representative confocal images of *ASN1-GFP AND ASN1-GFP URA7*△ strains in stationary phase. Scale bar 5 μm. (B) Cytoophidium abundance in Asn1-GFP cells is plotted along with the abundance of cytoophidia in cells with *URA7* knockout. (C) Asn1 cytoophidium length measurement without and with *URA7* deletion in stationary phase. (D) Western blot analysis of Asn1 protein level in *ASN1-GFP AND ASN1-GFP URA7△* strains at stationary phase. (E) Protein levels were plotted after normalization over alpha-actin levels and the protein level of Asn1 in Asn1-GFP cells is referred to as 1.

To answer the question of whether Asn1 cytoophidium length is affected by Ura7 disruption, we constructed Asn1-GFP Ura7△ cells and used Asn1-GFP cells as the control strain. We collected cells in stationary phase (Figure 3A) and analyzed the effect of Ura7 deletion on Asn1 cytoophidium abundance, length and protein level. The quantification results show that Ura7 knockout had no impact on abundance of Asn1 cytoophidia during the three growth phases (Figure 3B). The average length of Asn1 cytoophidia without and with Ura7 knockout was not significantly different, being, respectively, 0.60 and 0.57 μm (Figure 3C). Meanwhile, the protein level of Asn1 in the two cell strains was similar (Figure 3, D and E). Taken together, these results reveal that Asn1 and Ura7 cytoophidia have divergent effects on each other.

### Disassembly of the Ura7 and Asn1 cytoophidia

With the growth phase transition from exponential to stationary, nutrients are gradually depleted. Ura7 cytoophidia and Asn1 cytoophidia start to form in the exponential phase and diauxic phase, respectively. The greatest abundance of both types of cytoophidia is during the stationary phase. This indicates that the level of nutrients in the culture medium has a close association with cytoophidium formation.

**Figure 4:**
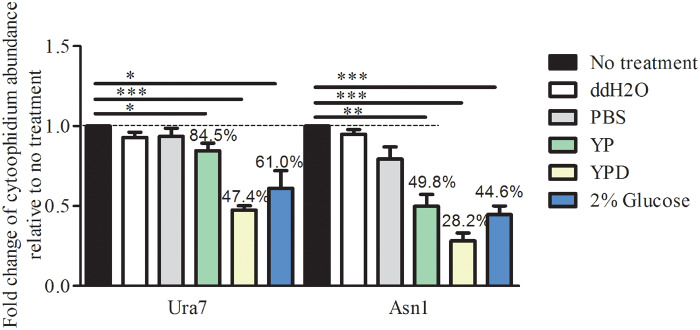
Ura7 and Asn1 cytoophidia respond to culture medium change. Cells were grown until stationary phase in rich medium which was then replaced with a different medium for 4 h: PBS, ddH_2_O, YP, YPD, or 2% glucose. The cells were collected and fixed by 4% PFA. Ura7 and Asn1 cytoophidium abundance was calculated by ImageJ Fiji software and plotted after three biological repeats. More than 200 cells were manually counted per strain per trial. *P < 0.05, **P < 0.01, and ***P < 0.001.

We compared the effects on Ura7 and Asn1 cytoophidium formation of changing to different culture media: ddH_2_O, PBS, YP, YPD, and 2% glucose (Figure 4). YPD is a nutritionally rich medium for the growth of *S. cerevisiae*, containing glucose, peptone and yeast extract. Peptone is a source of carbon, nitrogen, vitamins and minerals. Yeast extract supplies vitamins which stimulate bacterial growth. Glucose is the carbohydrate source.

Compared with no culture medium shift, ddH_2_O and PBS treatment had no significant effect. But, treatment with the other three media, YP, YPD and 2% glucose, resulted in significant disassembly of the Ura7 cytoophidia and Asn1 cytoophidia. The abundance of Ura7 cytoophidia decreased to 84.5%, 47.4%, and 60.1% with YP, YPD and 2% glucose, respectively. For Asn1 cytoophidia, it decreased to 49.8%, 28.2%, and 44.6%, respectively (Figure 4). Apparently, carbon source only or a mixture of carbon, nitrogen source and other nutrition facilitates the disassembly of Ura7 and Asn1 cytoophidia.

### Induction of Ura7 and Asn1 cytoophidia

Ura7 and Asn1 cytoophidium abundance reached a peak in the stationary phase. We wondered whether using stationary phase culture medium could induce cytoophidium formation in the other two growth phases. We first collected the stationary phase culture medium in which cells had been grown for one week and used it to replace the culture medium of exponential and diauxic phase cells for 4 hours. In the exponential growth phase, yeast cells utilize glucose as a carbon source to produce ethanol. When glucose is limiting, cells start to use the available ethanol as an energy source and enter into diauxic phase. In stationary phase, there is no cell division and yeast growth reaches a plateau.

**Figure 5:**
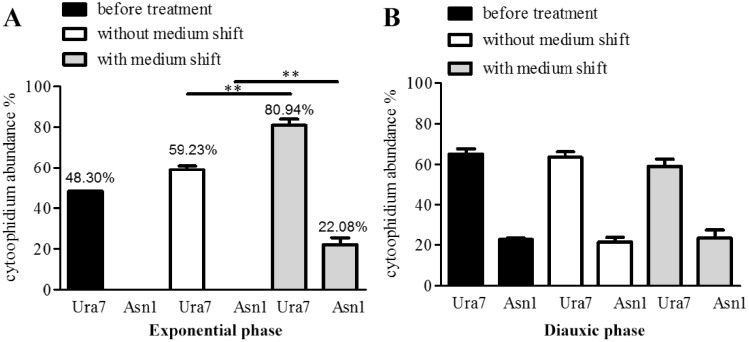
Stationary phase culture medium effect on Ura7 and Asn1 cytoophidia in exponential and diauxic phases. Stationary phase culture medium was collected after 168 h culture. Cells were grown in rich medium until exponential and diauxic phase. The medium was replaced with stationary phase culture medium when cells were at exponential phase and diauxic phase for 2 h. (A) Quantification of Ura7 and Asn1 cytoophidium abundance before medium shift in exponential phase along with the cells grown under no treatment and under medium shift. (B) The same analysis as in A was performed in diauxic phase. **P < 0.01.

According to the quantification results, the shift to stationary phase culture medium significantly increased both Ura7 and Asn1 cytoophidium formation in the exponential phase (Figure 5A), from 59.23% to 80.94% for Ura7 and 0% to 22.08% for Asn1. To our surprise, Ura7 and Asn1 cytoophidia in the diauxic phase were not influenced by this culture medium shift (Figure 5B).

### Formation of Asn1 foci in diauxic phase

**Figure 6:**
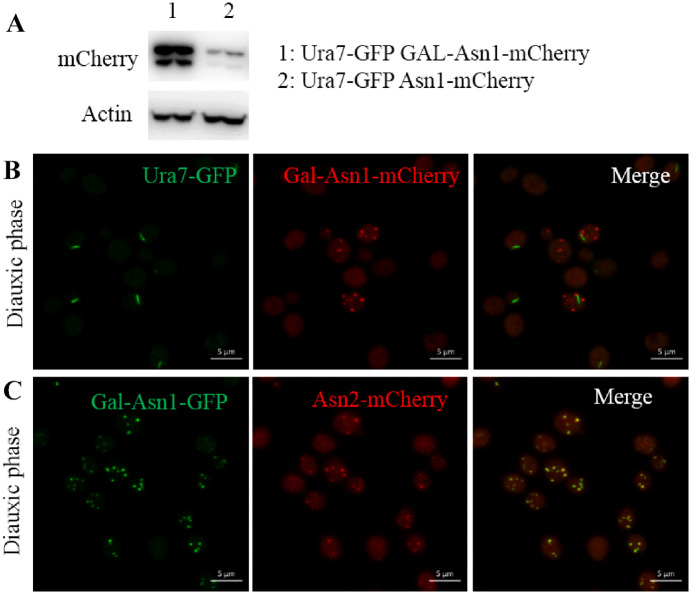
High Asn1 protein level results in Asn1 foci in diauxic phase. *URA7-GFP GAL-ASN1-MCHERRY* and *GAL-ASN1-GFP ASN2-MCHERRY* cells were grown in medium containing galactose. *URA7-GFP GAL-ASN1-MCHERRY* strain was generated from *URA7-GFP ASN1-MCHERRY* strain by inserting Gal promoter before the Asn1 coding sequence. The generation process of *GAL-ASN1-GFP ASN2-MCHERRY* strain was similar. (A) Asn1 protein level in *URA7-GFP GAL-ASN1-MCHERRY* and *URA7-GFP ASN1-MCHERRY* cells. (B) Representative images of *URA7-GFP GAL-ASN1-MCHERRY* cells in diauxic phase. (C) Representative images of *GAL-ASN1-GFP ASN2-MCHERRY* cells in diauxic phase. Scale bar: 5 μm.

Asn1 cytoophidia begin to appear in diauxic phase and increase in abundance from diauxic to stationary phase. It is reported that the protein level of CTP could regulate CTPS cytoophidium formation (Ingerson-Mahar *et al.* 2010; Chen *et al.* 2011). Hence, we increased the Asn1 protein level, by inserting the Gal promoter before the Asn1 coding sequence, to investigate the effect on Asn1 cytoophidium formation. The Western blot result shows that Asn1 protein level is dramatically elevated in Ura7-GFP Gal-Asn1-mCherry cells compared with Ura7-GFP Asn1-mCherry cells (Figure 6A). Besides, we found that Asn1 overexpression resulted in multi-dot foci in the diauxic phase (Figure 6B). We also observed a similar phenotype in Gal-Asn1-GFP Asn2-mCherry cells (Figure 6C). Meanwhile, we found that the distribution of Asn2 foci was the same as for Asn1 foci.

### A glutamine analog suppresses ASNS cytoophidium formation

**Figure 7:**
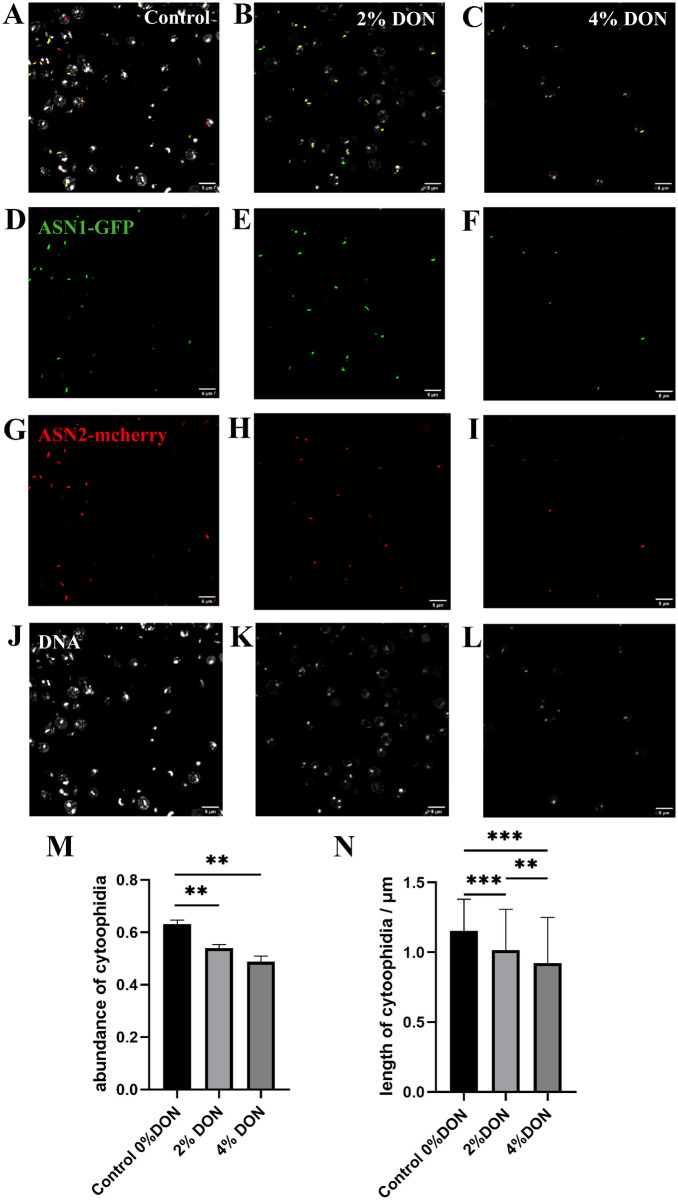
Glutamine analog suppresses ASNS cytoophidium formation. (A–C) ASN1-GFP ASN2-mcherry cells treated by 6-diazo-5-oxo-L-norleucine (DON), with concentrations at 0%, 2%, and 4%, respectively. (D–F) ASN1-GFP cytoophidia in ASN1-GFP ASN2-mcherry cells treated by DON, with different concentrations. (G–I) ASN2-mcherry cytoophidia in ASN1-GFP ASN2-mcherry cells treated by DON, with various concentrations. (J–L) DNA in ASN1-GFP ASN2-mcherry cells treated by DON. (M) The abundance of cytoophidia ASN1-GFP ASN2-mcherry cells treated by DON. (N) The average length of cytoophidia in ASN1-GFP ASN2-mcherry cells treated by DON. The experimental results were processed by ANOVA function of software GraphPad Prism. *P< 0.05, **P< 0.01, and ***P < 0.001.

Both CTPS and ASNS use glutamine as a nitrogen donor. In order to study the regulation of glutamine metabolism on the formation of ASNS cytoophidia, we used glutamine analog DON with various concentrations to treat ASN1-GFP ASN2-mcherry cells. The statistical results showed that the formation of ASNS cytoophidia was affected by the increase of DON concentration (Figure 7). Both ASN1 and ASN2 cytoophidia could be observed in more than 60% of cells without DON, and their average length was about 1.2 μm. When the DON content was 2%, the cytoophidia abundance was about 53%, and the average length was only about 1 μm. When the DON concentration was increased to 4%, the cytoophidia abundance was less than 50%, and the average length was less than 1 μM. These results suggest that DON has a negative effect on the formation of ASNS cytoophidia.

## Discussion

Awareness of the widespread presence of cytoophidia, the membraneless structures formed by metabolic enzymes, across species is greatly raised. The study of the relationship between cytoophidia and enzyme function attracts researchers’ attention. ASNS and CTPS are known to be glutamine-dependent metabolic enzymes that form cytoophidia. As the discovery of CTPS cytoophidia, intensive research in several species has been performed *in vivo* and *in vitro* (Barry *et al.* 2014; Daumann *et al.* 2018; Li *et al.* 2018; Zhou *et al.* 2019), and CTPS cytoophidia have been mentioned in other previous studies (Liu 2011; Gou *et al.* 2014; Zhang *et al.* 2014; Chang *et al.* 2015; Tastan and Liu 2015; Aughey and Liu 2016; Aughey *et al.* 2016; Liu 2016; Chang *et al.* 2017; Huang *et al.* 2017; Lynch *et al.* 2017; Chang *et al.* 2018; Lynch and Kollman 2020). It has been shown that the paralogs of ASNS (Asn1 and Asn2) are divergent in terms of cytoophidium formation (Zhang *et al.* 2018; Noree *et al.* 2019b), and our previous study also found that ASNS cytoophidia and CTPS cytoophidia have a spatial association. However, the understanding of ASNS cytoophidium formation and its regulation is still limited.

Why the filamentous structures containing CTPS or ASNS have not been reported before. In our opinion, there are at least three possible explanations (Liu 2011; Aughey *et al.* 2014; Liu 2016). Firstly, growth phases matter. In budding yeast, for example, the abundance of CTPS, ASNS, and glutamate synthase (GLT) changes from below 5% at exponential phase to above 70% at stationary phase (Shen *et al.* 2016). The filamentous structures of these proteins could have been ignored if the large-scale screening is carried at exponential phase. Secondly, some components in the culture medium have profound effects on the maintenance of cytoophidia. For example, the addition of glucose at the diauxic shift causes ASNS cytoophidium disassembly (Zhang *et al.* 2018). Thirdly, the way to prepare cells for microscopic examination can be tricky. In fission yeast, cytoophidia are very sensitive to temperature (Zhang and Liu 2019). If the cells are treated by cold phosphate buffer saline (a typical method) for a few minutes before fixation, cytoophidia will disappear in most, if not all, cells.

Here, we found that the formation of ASNS cytoophidia and CTPS cytoophidia shows a similar pattern in *S*. *cerevisiae*. For ASNS and CTPS, the process begins in the exponential phase and diauxic phase, respectively, and reaches a peak in the stationary phase. There is a difference in morphology as CTPS cytoophidia are crooked and ASNS cytoophidia are stick shaped. The average length of CTPS cytoophidia is more than twice as long as ASNS cytoophidia, and CTPS cytoophidia have a wider length range than ASNS cytoophidia. The responses of the two types to medium switching is similar. A recent study from our lab shows that hypoosmolality disrupts cytoophdium integrity during nitrogen starvation (Li and Liu 2020).

Our work also shows that disruption of either Asn1 or Asn2 shortens the average length of Ura7 (*i.e.*, CTPS) cytoophidia, which suggests that there is interaction between these different types of cytoophidia. In addition, features of cytoophidium formation and its pattern in response to growth phase and medium change were studied. The cells in stationary phase were stressed by the lack of nutrients. We find that the stationary phase culture medium shift increases the abundance of Ura7 cytoophidia and triggers the formation of Asn1 cytoophidia, suggesting that nutrient deprivation has an effect on cytoophidium formation.

We previously showed that treatment of a glutamine analog DON promotes CTPS cytoophdium assembly in fruit fly and human cells (Chen *et al.* 2011), as well as in fission yeast (Zhang and Liu 2019). In the contrary, DON treatment induces the disassembly of CTPS filaments in bacteria (Ingerson-Mahar *et al.* 2010). Here we find that ASNS cytoophidia also respond to the treatment of DON. However, DON treatment seems suppressing the formation of ASNS cytoophidia. Together, these results suggest that the formation and maintenance of CTPS and ASNS cytoophidia link to glutamine metabolism. As both CTPS and ASNS use glutamine as a substrate, it would be interesting to analyze the dynamic behavior of CTPS cytoophidia and ASNS cytoophidia with or without DON treatment. Further studies are required to increase our understanding of the relationship between ASNS cytoophidia and CTPS cytoophidia.
